# Telemedicine-Assisted Work-Related Injuries Among Seafarers on Italian-Flagged Ships: A 13-Year Retrospective Study

**DOI:** 10.3390/healthcare13182375

**Published:** 2025-09-22

**Authors:** Getu Gamo Sagaro, Francesco Amenta

**Affiliations:** 1 Centro Internazionale Radio Medico (C.I.R.M.), Research Department, 00144 Rome, Italy; francesco.amenta@unicam.it; 2 School of Public Health, College of Health Sciences and Medicine, Wolaita Sodo University, Wolaita Sodo P.O. Box 138, Ethiopia

**Keywords:** seafarers, ships, accidents, work-related accidents, occupational injuries, epidemiology

## Abstract

**Background**: Seafarers are highly susceptible to work-related injuries, which can result in serious consequences or permanent disabilities. Understanding the frequency and characteristics of occupational injuries is crucial for developing effective prevention strategies and identifying their underlying patterns and causes. This study aimed to determine the frequency and characteristics of telemedicine-assisted work-related injuries among seafarers on board Italian-flagged vessels. **Methods**: A retrospective descriptive study was conducted to analyze occupational injuries using medical data recorded in the Centro Internazionale Radio Medico (C.I.R.M.) database from 1 January 2010 to 31 December 2022. Injuries in the database were coded according to the 10th revision of the International Classification of Diseases (ICD-10) by the World Health Organization (WHO). Variables extracted from the database included injury type, seafarers’ age, rank, nationality, worksite, gender, date of injury, affected body region, clinical outcomes, and other demographic and occupational characteristics. Injury frequency and characteristics (e.g., location, type, and cause) were analyzed and stratified by seafarers’ rank and worksite groups. **Results**: The analysis included 793 seafarers who sustained injuries. Their average age was 39.15 ± 10.49 years (range: 21 to 70 years). Deck ratings and engine officers accounted for 27.9% and 20% of those who claimed injuries, respectively. 39.2% of injured seafarers were aged between 30 and 40 years. In terms of affected body parts, the most reported injuries were to the hand/wrist (33.3%), followed by the knee/lower legs (21%), and the head/eye (19%). Open wounds (38%) and burns/abrasions (14%) were the most common types of injury. Slips/falls (32%), burns/explosions (16.6%), and overexertion while lifting or carrying (14.8%) were the leading causes of injury during the study period. Nearly 35% of injuries affected workers on the deck and were due mainly to slips/falls, 19% in the engine room were due to being caught in machinery or equipment, and 32.5% in the catering department were due to burns/explosions. **Conclusions**: One-third of seafarers who suffered work-related injuries sustained hand and/or wrist injuries, with slips/falls being a significant cause. The results of this study emphasize the need for preventative measures in the marine sector, particularly to reduce risks associated with slips and falls, overexertion, and other injury-causing factors. Campaigns for the larger use of protective equipment are desirable to reduce occupational accidents at sea and provide better health protection for seafarers.

## 1. Introduction

Work-related injuries are defined as any damage to the body caused by energy transfer during work, occurring shortly after exposure [1]. These injuries can result from acute exposure to physical and chemical factors. They also arise from sudden deprivation of essential agents like oxygen or heat and are distinct from occupational diseases. In general, occupational injuries happen suddenly due to exposure to harmful conditions at work (like accidents, shocks, or burns). On the other hand, occupational diseases develop gradually over time due to prolonged or repeated exposure to physical, chemical, biological, or psychosocial hazards in the workplace, such as musculoskeletal disorders, respiratory diseases, hearing loss, etc.

Seafarers operate in a hazardous environment that includes physical, ergonomic, chemical, biological, psychological, and social factors, which may lead to work-related injuries and diseases [2]. Workers at sea face higher rates of mortality, injuries, and diseases compared to those working ashore [3]. They face occupational hazards specific to their environment, including the challenges of living and working in isolated and confined spaces, performing physically demanding tasks, such as cargo handling and ship maintenance, and dealing with environmental risks like extreme weather conditions and exposure to heavy machinery. Seafarers have a one in eleven chance of being injured while on duty, with physical injuries often being severe and a leading cause of disability [4]. According to a study conducted on British merchant ships between 2003 and 2012, the shipping fatal accident rate was 21 times higher than that of the general British workforce, 4.7 times higher than that of the construction industry, and 13 times higher than that of the manufacturing industry [5]. The death rate among Danish seafarers aboard was 11.5 times higher than the death rate among Danish male workers ashore [6]. These figures suggest that seafarers are particularly vulnerable to harm in the workplace, underscoring the need for increased efforts to protect them.

Studies conducted onboard ships reported that work-related injuries accounted for most medical events requiring teleconsultation. For instance, a recent study on German-flagged container ships found that accidents were the second most frequent reason for seeking medical advice, accounting for 17.9% of cases [7]. The results of our previous study also indicated that work-related injuries constituted the second most common reason for accessing teleconsultation onboard (16.4%) between 2010 and 2018 [8]. A study conducted among American seafarers reported that injuries most frequently resulted in lost work compared to diseases [9]. According to a study of Swedish merchant and passenger ships between 1997 and 2012, 20% of all contacts for medical advice resulted from accidents involving seafarers [10]. Occupational injuries are consistently reported as a major reason for seeking telemedical consultations on board ships. However, existing research has primarily focused on the frequency of injuries, with limited attention to the detailed characteristics of those injuries. The difference in injury patterns between different occupational and worksite groups on ships remains largely unexplored.

The present study has analyzed the frequency and characteristics of telemedicine-assisted work-related injuries among seafarers on board Italian-flagged ships from 2010 to 2022. By evaluating differences in injury patterns across occupational and worksite groups, the study provides valuable insights for the development of targeted preventive measures, improving telemedical assistance to seafarers, and enhancing maritime occupational safety.

## 2. Materials and Methods

A retrospective descriptive approach to analyze medical data obtained from the database of the Centro Internazionale Radio Medico (C.I.R.M.) was followed. C.I.R.M. is the Italian Telemedical Maritime Assistance Service (TMAS), offering remote medical support to seafarers and passengers on board ships without a doctor since 1935. The service covers 24 h a day, 7 days a week, and 365/366 days a year, regardless of seafarers’ nationality and vessel positions. Over the past 90 years, C.I.R.M. has assisted more than 140,000 individuals at sea, conducting over 700,000 teleconsultations, an average of five per patient.

The present study has considered all seafarers assisted onboard Italian-flagged vessels for work-related injuries from 1 January 2010 to 31 December 2022. A total of 960 seafarers reported injuries during the study period and received medical assistance from C.I.R.M. through various telemedicine methods, including video calls, emails, and radio or cell phone communications. After assisting a patient with the reported injury, a trained C.I.R.M. telemedicine data manager routinely extracted the patient’s demographic information, injury causes, diagnoses, affected body parts, end-of-follow-up outcomes, number of teleconsultations, and other medical data. Occupational injuries were recorded in the database following the ICD-10 classification by the World Health Organization, covering chapter XIX codes S00-S99 and T00-T98. For this study, data on individual injured seafarers, including age, sex, occupation, nationality, work sites, places where the injury occurred (in ports or at sea), parts of the body affected, nature of injuries, causes of injury, number of consultations, and outcomes, were systematically retrieved from the database.

Data were checked, recoded, entered, and analyzed using the R programming language (Version 4.4.1) [11]. We calculated a seafarer’s age by subtracting his or her date of birth from the date on which medical advice was given. We excluded seafarers without a recorded birth date from the study. Seafarers were classified according to their occupational rank into deck officers, deck ratings, engine officers, engine ratings, galley staff, and others, while worksites were categorized into deck, engine, and catering departments. Seafarers were classified into four age groups: under 30 years, from 30 to 40 years, from 41 to 50 years, and from 51 years and older. Injury characteristics were categorized by type, cause, and anatomic location. A descriptive statistical analysis of seafarers’ demographic data was performed to evaluate work-related injury distribution. Differences in the distribution and characteristics of injuries were examined and compared across seafarers’ occupational ranks and worksite groups using descriptive statistics. Comparison of categorical variables was performed using the chi-square test to determine if the observed distribution significantly differs from the expected under the assumption of independence, considering a *p*-value below 0.05 as significant.

## 3. Results

A total of 793 subjects with work-related injuries were included in the analysis, out of 960 seafarers suffering from accidents assisted in Italian-flagged ships from 2010 to 2022. 17.4% (167) of injured seafarers were excluded from analysis due to incomplete relevant information, such as missing age data. The mean age of seafarers at injury was 39.15 ± 10.49 years (range: 21 to 70 years). Nearly two-fifths of seafarers who reported injuries were aged between 30 and 40 years (39.2%), while 22.8% were aged between 41 and 50 years. Most seafarers who reported injuries were deck ratings (27.9%), followed by engine ratings (20.3%) and engine officers (20.1%). Regarding their workplace, 46.3% of seafarers who reported injuries were from the deck department, while 40.4% were engine department employees.

The nationality of 680 injured seafarers (85.7% of all reported injuries) was also considered. As a result, 62.2% of sailors were non-European, while 30.3% were Italian. Indian and Filipino sailors made up 54.2% and 37.8% of the non-European seafarers consulted for injuries, respectively. According to these statistics, among the non-European workers, Indian seafarers were those most assisted. The distribution of injuries by nationality was statistically significant (X^2^ = 308.1, *p*-value < 0.001). In other words, injuries were not equally distributed among Italians, other Europeans, and non-Europeans. In terms of the place where injuries occurred, we retrieved data from 588 contacts (74% of all reported injuries). 77.6% of accidents occurred at sea, while the remaining 22.4% occurred in port. Almost all seafarers assisted with injuries were male (96%) (Table 1).

### 3.1. Number of Reported Injuries (%) Between 2010 and 2022

**Figure 1:**
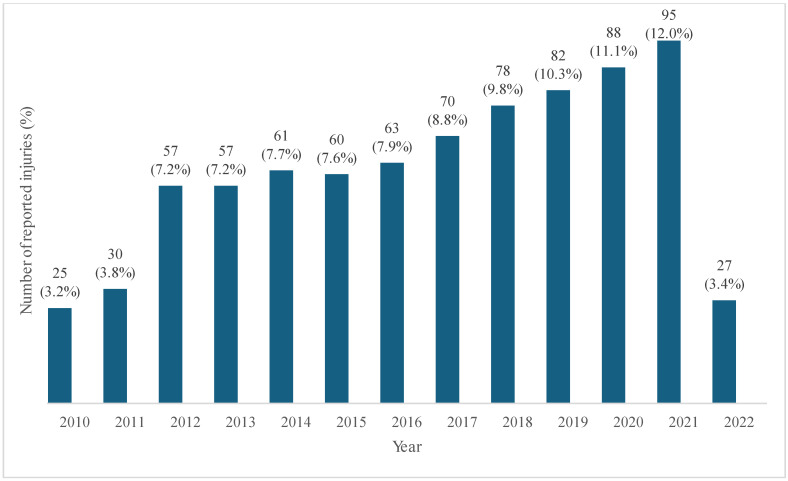
Number of reported injuries (%) from Italian-flagged vessels from 2010 to 2022.

As shown in Figure 1, the percentage of work-related injuries remarkably increased in 2012 compared to the previous years. After stabilization from 2012 to 2016, the frequency of reported injuries showed an increase up to 2021 (Figure 1).

### 3.2. Distribution of Injuries by Anatomic Location

**Figure 2:**
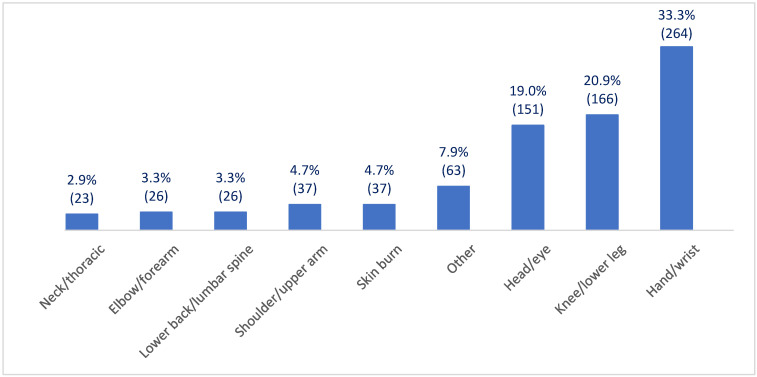
Frequency of work-related injuries by anatomic location on Italian-flagged ships from 2010 to 2022.

Injury analysis based on the anatomic location of body parts revealed that the most common injuries affected hand and/or wrist injuries. These issues were responsible for 33.3% of all reported injuries during the study period. The second most frequently affected body parts were the knee and/or lower legs, accounting for 21% of reported cases. Head and/or eye injuries were the third most common injuries, accounting for 19% of cases assisted. Figure 2 summarizes the distribution of injuries by anatomic location on Italian-flagged ships between 2010 and 2022 (Figure 2).

### 3.3. Types, Causes, and Location of Injuries by Rank Group of Seafarers

**Figure 3:**
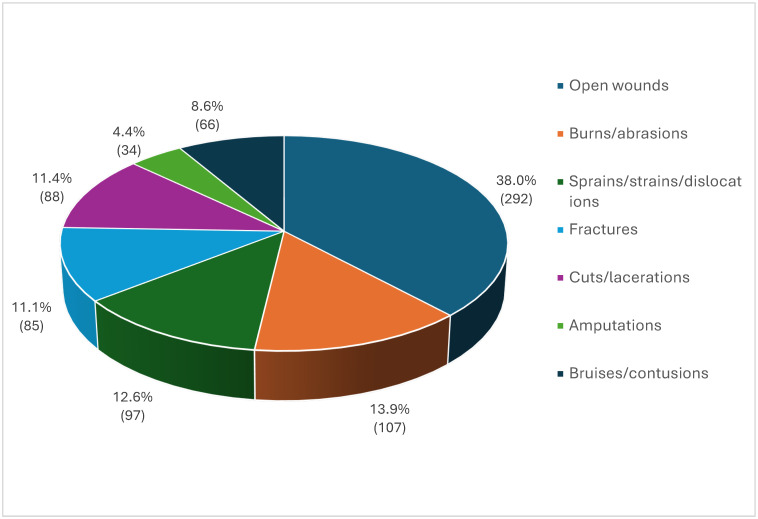
Types of injuries on Italian-flagged vessels, 2010–2022.

The types of injuries were analyzed in 769 contacts (97% of all reported injuries). Open wounds were the most frequently reported types of injuries (38%), followed by burns/abrasions (14%) and sprains/strains/dislocations (12.6%), which all together represented more than three-fifths of all reported injuries (Figure 3).

**Figure 4:**
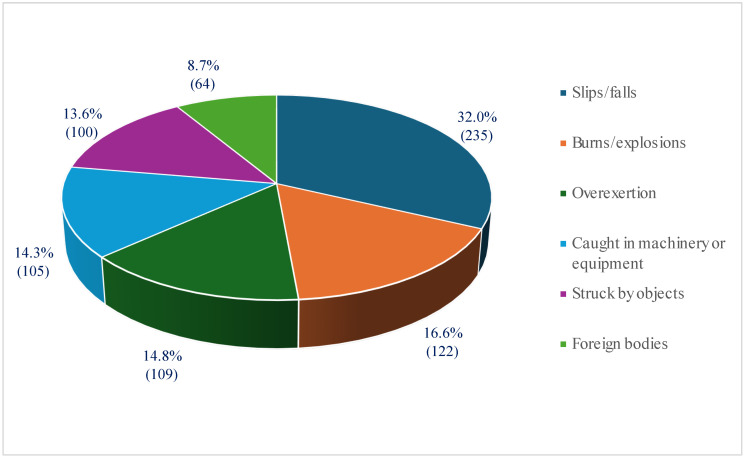
Causes of injuries on Italian-flagged vessels, 2010–2022.

Causes of accidents were analyzed in 735 cases (92.7% of all reported injuries). Slips/falls were the leading cause of injuries, accounting for 32%, followed by burns/explosions (16.6%) and overexertion while lifting or carrying items (14.8%) (Figure 4).

The data of the analysis of the occurrence of accidents per occupational group are summarized below.

(1) Deck ratings: Most injuries occurred in the hand/wrist (32%), followed by the knee/lower legs (25.4%), and the head/eye (20%). (2) Deck officers: Most injuries affected, in descending order, hand/wrist (32.2%), head/eye (22.6%), and knee/lower leg (21.2%). (3) Engine ratings: Most injuries affected the hand/wrist (34.8%), head/eye (23.6%), and knee/lower leg (19.3%). (4) Engine officer: Most injuries occurred in the hand/wrist (35.8%), head/eye (17.6%), and knee/lower leg (14.5%). (5) Galley staff: Most injuries affected the hand/wrist (35%), skin (12.5%), and knee/lower legs (13.8%) (Table 2).

Open wounds (41.2%) occurred more often in deck ratings, whereas sprains/strains/dislocations (12%) and fractures (14.4%) were most frequent in deck officers. Burns/abrasions (27.5%) and bruising/contusions (10%), types of injuries most frequently reported by galley staff. Cuts/lacerations (17.4%) were the most common types of injury in engine ratings, while amputations (7%) were in engine officers (Table 2). Slips/falls (35.3%) and overexertion while lifting or carrying items (19.5%) were the leading causes of injuries among deck ratings, while being caught in machinery or equipment was the major cause of injuries among engine officers (19%). Burns/explosions were the most common causes of injuries among galley staff (Table 2). Significant differences in the distribution of injury location (*p* < 0.01), type (*p* < 0.001), and injury cause (*p* < 0.001) were noticeable between the different rank groups.

### 3.4. Injuries by Worksites of Seafarers

In our study, vessel areas where accidents occurred were divided into three departments: deck, engine, and catering. Most injuries were reported from the deck (47.8%), followed by the engine (41.7%) and catering (10.4%) departments. Most hand/wrist injuries (35.3%) were reported from the engine department, while knee/lower leg injuries (23.7%) were reported from the deck department. 12.5% of skin burn injuries occurred in the catering department. The three most common types of injury in the deck department were, in the order, open wounds (40%), sprains/strains/dislocations (15%), and fractures (13%), while the catering department reported burns/abrasions (27.5%), cuts/lacerations (13.8%), and bruises/contusions (10%). The leading causes of injury in the deck departments were slips/falls (35%) and overexertion while lifting or carrying items (19%), whereas being caught in machinery or equipment (19%) and being struck by objects (15%) were the most common causes of injuries in the engine department. Burns/explosions (32.5%) and foreign bodies (12.5%) were common causes of injury in the catering department (Table 3). Work sites exhibited significant differences in the distribution of injury location (*p* < 0.001), type (*p* < 0.01), and cause (*p* < 0.001).

## 4. Discussion

From 2010 to 2022, C.I.R.M. has assisted 960 seafarers on board Italian-flagged vessels due to work-related injuries. These assistances required 4320 teleconsultations, with an average of 4.5 teleconsultations per seafarer. In this study, we included 793 individuals with work-related injuries out of the 960 seafarers assisted. The reason for their exclusion was that they did not meet the inclusion criteria.

Occupational injuries are the leading cause of work absence, mortality, and medical unfitness for work at sea among sailing seafarers [9,12,13]. Limited research has examined the variations in the frequency and characteristics of work-related injuries among different seafarer occupational groups and work sites. The current study has analyzed data from the C.I.R.M. database on telemedicine-assisted work-related injuries sustained on Italian-flagged vessels between 2010 and 2022, examining differences in injury frequency and characteristics based on seafarers’ roles and worksite groups. We have observed that most deck ratings (28%) reported work-related injuries compared to other occupational groups. This might be due to the nature of their duties. Deck ratings are typically responsible for physically demanding and labor-intensive tasks such as cargo handling, mooring operations, maintenance work, cleaning, and assisting with navigation-related activities. About two-fifths of study participants who reported injuries were aged between 30 and 40 years (39.2%). This relatively young age of people experiencing accidents suggests a lack of experience working at sea. Our findings are consistent with those of a recent study on German-flagged ships, which reported that most deck ratings sustained injuries (22.5%) [7]. The reason is probably because deck ratings often perform physically demanding tasks, such as loading and unloading cargo, which increases their risk of injury. Further, they frequently work in challenging conditions, including rough seas and adverse weather conditions, which can lead to accidents. Moreover, long working hours and fatigue may further increase the likelihood of injuries among this group [7].

Injury analysis based on the anatomic location of body parts revealed that the most common injuries affected, in descending order, hand/wrist (33.3%), knee/lower legs (21%), and head/eye (19%). These injuries together represented three-quarters of all reported injuries during the study period. The percentage distribution of injuries across the body suggests a higher frequency of hand and/or wrist injuries compared to other regions, reflecting the nature of work on ships, which often requires repetitive movements or handling of heavy equipment. In line with our findings are the results of a study performed on American seafarers reporting that the most frequently injured body parts were the upper extremities (34%), particularly hand and wrist injuries [9]. Moreover, the above study on German-flagged ships found that hands (44%) and head/neck (36.3%) accounted for most of all injuries [7]. Safety training programs that emphasize proper lifting techniques and the use of protective wear are crucial to reducing the occurrence of these common injuries. Maintaining equipment regularly and adhering to all safety protocols can also help mitigate risks. Moreover, promoting a culture of safety and awareness of prevention among crew members may contribute to a safer working environment aboard a vessel.

Regarding causes of injury, slips/falls (32%), burns/explosions (16.6%), and overexertion while lifting or carrying heavy weights (14.8%) were the leading causes of injuries on board Italian-flagged vessels during the study period, collectively contributing to nearly two-thirds of all reported injuries. An investigation on fisheries revealed that falls and accidents related to machines were the most common causes of occupational injuries [14]. Accidents related to accessing and boarding ships often involve the angle of inclination of stairways, gangways, ladders, and other boarding locations [15]. 44% of injuries among seafarers were related to slips, falls, and trips (STFs), as reported by Jensen OC et al. in 2001 [16]. Furthermore, in 2005, it was reported that 43% of injuries were related to STFs [17]. These findings are consistent with those of the International Labor Organization (ILO), reporting that 35% of injuries were related to STFs [18]. To prevent slips and falls, it is essential to maintain clean, dry surfaces and ensure that wet areas are marked. The implementation of regular safety training can help crew members avoid overexertion injuries caused by heavy weights. In addition, installing fire suppression systems and providing adequate personal protective equipment can reduce the risk of burns and explosions.

In terms of the nature of injury, open wounds were the most common type, representing approximately 38% of all reported injuries. This prominence can be explained by the nature of shipboard tasks, which often involve the use of sharp materials, heavy machinery, and direct manual handling of equipment and cargo. Operations on deck and aboard machinery expose seafarers to risks such as cuts from cables and ropes, punctures from tools or machinery, and lacerations caused by slipping or contact with hard surfaces. Burns or abrasions accounted for 16.6% of the total reported injuries, followed by dislocations, sprains, or strains at 12.6%. Our findings align with a study on German-flagged container ships, which reported that the most common types of injuries were open wounds (33.3%) and burns (23.3%) [7]. In many workplaces, accidents involving sharp objects, machinery, or tools often result in open wounds. Furthermore, slips and falls may result in cuts and lacerations, contributing to the high rate. This type of injury is also more likely to occur when there are inadequate safety measures and insufficient protective equipment. It is important to note that these injuries can lead to serious health complications, such as infection and nerve damage. To minimize the risk of such injuries, proactive measures are essential.

Occupational rank-related differences in injury location: hand/wrist injuries were more common among engine officers (35.8%), head/eye injuries among engine ratings, knee/lower leg injuries among deck ratings, shoulder/upper arm injuries among deck officers, and skin burn injuries among galley staff members. As a result of the specific tasks and environments associated with each role, these differences may exist. Frequently, engine officers work with machinery, increasing their risk of hand/wrist injuries, while engine ratings are exposed to conditions that are likely to cause head/eye injuries. Deck officers and deck ratings perform physical tasks on deck, which may explain the prevalence of knee/lower leg and shoulder/upper arm injuries. Similarly, galley staff are regularly exposed to heat and cooking equipment, increasing their risk of skin burns. It is possible to reduce these occupational injuries by implementing targeted safety measures and training programs. It is recommended that engine officers be provided with protective gloves and undergo regular safety drills on how to handle machinery. Engine ratings should wear protective eyewear to prevent head and eye injuries. Deck officers and ratings may benefit from ergonomic tools and proper lifting techniques to avoid shoulder and knee injuries. Moreover, galley staff should have access to heat-resistant gloves and equipment to minimize skin burn risk.

The variation in types of injuries observed across different rank groups can be largely attributed to the nature of job roles and daily activities on board. Each group performs distinct tasks that expose them to specific risks, which in turn influence the pattern of reported injuries. For instance, open wounds were more common among deck ratings (41.2%), dislocations/sprains/strains were among deck officers (20%), burns/abrasions were among galley staff members (27.5%), cuts/lacerations were among engine ratings (17.4%), and amputations were among engine officers (7%). Our findings are consistent with those of the study conducted on German-flagged container ships, which reported open wounds (33.3%) and burns (23.3%) as the most common injuries [7].

The above data confirm that seafaring is one of the most hazardous occupations, and crew members face various challenges due to their working environment. For instance, the floors, stairs, ladders, doors, and gaps can be dangerous, especially during storms and rain due to the wetness. Our study has shown that 35.5% of the reported injuries in deck ratings resulted from slips/falls, 32.5% were related to burns/explosions in galley staff, and nearly 19% were related to being caught in machinery and equipment by engine officers and engine ratings. To prevent these issues, safety protocols should be improved in specific areas to reduce workplace injuries. It is evident from the high rate of slips and falls among deck ratings that better traction surfaces and fall prevention training are needed. Furthermore, the significant number of burns and explosions among galley employees underscores the importance of implementing stricter safety measures and training programs to ensure the safe handling of hazardous materials and equipment.

Analysis of work site-related differences in injury location revealed that most hand/wrist (35.3%) and elbow/forearm (4.1%) injuries occurred in the engine departments of ships, while most head/eye (21%), knee/lower legs (24%), and shoulder/upper arm (6%) were reported from the deck department. Open wounds (40%), fractures (13%), and dislocations/sprains/strains (15%) were the most common issues in the deck department, while cuts/lacerations (13.4%) and amputations (6.3%) were the most common injury natures in engine departments. In the catering departments, burns/abrasions (27.5%), cuts/lacerations (13.8%), and bruises/contusions (10%) were the most common types of injury. Most deck department injuries were caused by slips/falls (35%) and overexertion while lifting and carrying (19%). In engine departments, more frequent accidents were due to being caught in machinery or equipment (19%) and struck by objects (14.7%). In catering areas, burns/explosions (32.5%) were the main cause of accidents. In the deck department, injuries often resulted in fractures and dislocations, indicating a higher severity level compared to other departments. In the engine department, a higher incidence of amputations and severe injuries, often due to machinery-related accidents, was noticeable. Catering department workers primarily dealt with less severe injuries such as burns and bruises, reflecting less relevant risks in that ship area. In general, different ship areas are exposed to injuries of different types and severity. This indicates that safety protocols must be tailored to the specific risks of each department.

Telemedicine plays an increasingly essential role in addressing work-related injuries among remote populations, particularly seafarers who often work far from immediate medical support [19,20]. Seafarers frequently sustain injuries in environments where access to medical facilities is delayed due to long voyages or remote locations. Telemedicine enables real-time consultation with shore-based doctors or healthcare providers, allowing for early diagnosis, timely intervention, and appropriate triage of injuries [20,21]. This reduces complications from delayed treatment and ensures a safer return to work. In terms of severe injuries, telemedical guidance can optimize first aid, stabilize the patient, and determine whether emergency evacuation is necessary [21]. Similarly, it reduces the overall health burden by minimizing injury severity and long-term disability. In terms of international regulations and standards, the use of telemedicine in maritime healthcare is supported by international conventions and guidelines. As for the guidelines, the International Labor Organization’s (ILO) Maritime Labor Convention (MLC, 2006) and the International Maritime Organization’s (IMO) International Convention on Standards of Training, Certification and Watchkeeping for Seafarers (STCW) require that ships ensure access to medical care equivalent to that available on shore [2,22]. In terms of standards, the World Health Organization (WHO) and the European Maritime Safety Agency (EMSA) recognize telemedical maritime assistance services (TMAS) as a standard means of fulfilling these obligations [19,23]. This regulatory framework not only legitimizes telemedicine but also mandates its integration into occupational health systems at sea, reinforcing its role in injury prevention and management. Most of the TMAS centers, including C.I.R.M., offer free medical assistance to ships during emergency consultations, whether related to injuries or illnesses. However, occupational injuries impose a substantial financial burden on shipping companies through direct medical costs, emergency evacuations, lost working days, compensation claims, and potential legal liabilities [24]. Telemedicine helps reduce these costs by reducing the frequency of unnecessary evacuations or diversions of the ship, shortening recovery times through early intervention, and preventing minor injuries from escalating into severe or chronic conditions [19,20,21].

Limitations of the study: One of the main limitations of this study is the lack of detailed demographic, occupational, and exposure-related information for the total at-risk population of seafarers working on Italian-flagged ships. Consequently, it was not possible to identify predictors of work-related injuries or use more advanced statistical models. Instead, the analysis was limited to describing injuries based on their characteristics, such as type, location, and cause. To fill this gap, future studies should systematically include demographic and occupational variables, as these factors may significantly influence injury risks and outcomes. Another limitation is the very low representation of female seafarers in the dataset, making up only 3.9% of reported injuries. This prevented us from making meaningful comparisons based on sex. Given the growing global interest in gender diversity in maritime professions, future research should focus more on the experiences and risks faced by female seafarers, even if their numbers remain relatively low. Additionally, the retrospective design of the study limited the analysis to variables already available in the dataset, which may not fully capture the complexity of work-related injury risks on board. Therefore, prospective studies with more comprehensive data collection are recommended to gain deeper insights. Despite these limitations, this study stratified injuries by mechanism, providing valuable data to identify priority areas of concern. These findings can inform maritime health policymakers, employers, and other stakeholders in developing targeted prevention strategies. Integrating these data into occupational health and safety programs is essential for reducing the burden of injuries among seafarers and improving the overall safety culture in the maritime industry.

## 5. Conclusions

In summary, hand and/or wrist injuries were the most frequently occurring injuries on Italian-flagged ships between 2010 and 2022, followed by knee and/or lower leg injuries and head and/or eye injuries. Slips/falls, burns/explosions, and overexertion while lifting or carrying were the leading causes of injury during the study period. In the deck department, slips/falls were the leading causes of injuries. In the engine department, most of the injuries were related to being caught in machinery or equipment, and in the catering department, most of the injuries were due to burns/explosions. These findings indicate the need for continued focus on preventive measures, safety equipment, and training to minimize the frequency of injuries and ensure a safe working environment for all crew members. Slip/fall incidents can be significantly reduced by implementing non-slip surfaces and conducting regular safety drills within the deck department. Proper guarding of machinery and routine maintenance checks can prevent injuries caused by equipment caught in engine departments. Catering departments can reduce the risk of burns and explosions by providing fire-resistant protective gear and comprehensive training on handling hot surfaces and flammable materials. To improve understanding of risk factors, future studies are recommended to adopt prospective approaches that allow for a more precise assessment of injury predictors. This would allow the application of advanced statistical models to establish causal relationships rather than simply describing injury patterns.

## Figures and Tables

**Table 1:** Demographic and other characteristics of seafarers with work-related injuries on Italian-flagged vessels between 2010 and 2022.

Variable	Frequency (%)
**Age group (years)** (n = 793)	
<30	167 (21.1)
30–40	311 (39.2)
41–50	181 (22.8)
51+	134 (16.9)
**Gender** (n = 793)	
Male	762 (96.1)
Female	31 (3.9)
**Rank** (n = 793)	
Deck officer	146 (18.4)
Engine officer	159 (20.1)
Deck rating	221 (27.9)
Engine rating	161 (20.3)
Galley	80 (10.1)
Other	26 (3.3)
**Nationality** (n = 680)	
Italian	206 (30.3)
Other European	51 (7.5)
Non-European	423 (62.2)
**Vessel department** (n = 793)	
Deck	367 (46.3)
Engine	320 (40.4)
Catering	80 (10.1)
Other	26 (3.3)
**Vessel location** (n = 588)	
Sea	456 (77.6)
Port	132 (22.4)

**Table 2:** Characteristics of injury by rank group of seafarers on Italian-flagged vessels from 2010 to 2022.

	Deck Rating (No. 221)	Deck Officer (No. 146)	Engine Rating (No. 161)	Engine Officer (No. 159)	Galley (No. 80)
	No. (%)	No. (%)	No. (%)	No. (%)	No. (%)
**Anatomic location** (n = 767)					
Hand/wrist	71 (32)	47 (32.2)	56 (34.8)	57 (35.8)	28 (35)
Head/eye	44 (19.9)	33 (22.6)	38 (23.6)	28 (17.6)	5 (6.3)
Knee/lower leg	56 (25.4)	31 (21.2)	31 (19.3)	23 (14.5)	11 (13.8)
Skin burn	6 (2.7)	2 (1.4)	9 (5.6)	9 (5.7)	10 (12.5)
Shoulder/upper arm	12 (5.4)	10 (6.8)	2 (1.2)	10 (6.3)	2 (2.5)
Lower back/lumbar spine	7 (3.2)	4 (2.7)	5 (3.1)	6 (3.8)	4 (5.0)
Neck/thoracic	5 (2.3)	8 (5.5)	4 (2.5)	2 (1.3)	2 (2.5)
Elbow/forearm	6 (2.7)	4 (2.7)	4 (2.5)	9 (5.7)	3 (3.8)
other	14 (6.3)	7 (4.8)	12 (7.5)	15 (9.4)	15 (18.8)
**Types of injury** (n = 746)					
Open wound	91 (41.2)	56 (38.4)	57 (35.4)	58 (36.5)	23 (28.8)
Burn/abrasions	25 (11.3)	8 (5.5)	23 (14.3)	24 (15.0)	22 (27.5)
Sprains/strains/dislocations	26 (11.8)	29 (19.9)	14 (8.7)	17 (10.7)	9 (11.3)
Fracture	27 (12.2)	21 (14.4)	16 (9.9)	17 (10.7)	2 (2.5)
Bruise/contusions	19 (8.6)	11 (7.5)	13 (8.0)	11 (7.0)	8 (10.0)
Cut/lacerations	20 (9.0)	12 (8.2)	28 (17.4)	15 (9.4)	11 (13.8)
Amputation	10 (4.5)	1 (0.7)	9 (5.6)	11 (6.9)	2 (2.5)
**Causes of injury** (n = 724)					
Slips/falls	78 (35.3)	50 (34.2)	43 (26.7)	46 (28.9)	14 (17.5)
Burn/explosions	26 (11.8)	11 (7.5)	30 (18.6)	25 (15.7)	26 (32.5)
Caught in machinery or equipment	26 (11.8)	10 (6.8)	30 (18.6)	30 (18.9)	8 (10.0)
Struck by objects	29 (13.1)	20 (13.7)	19 (11.8)	28 (17.6)	3 (3.8)
Overexertion	43 (19.5)	26 (17.8)	15 (9.3)	15 (9.4)	9 (11.3)
Foreign bodies	12 (5.4)	15 (10.3)	15 (9.3)	12 (7.5)	10 (12.5)

**Table 3:** Characteristics of injury by worksite on Italian-flagged vessels from 2010 to 2022.

	Deck (No. 367)	Engine (No. 320)	Catering (No. 80)
	No. (%)	No. (%)	No. (%)
**Anatomic location** (n = 767)			
Hand/wrist	118 (32.2)	113 (35.3)	28 (35.0)
Head/eye	77 (21.0)	66 (20.6)	5 (6.3)
Knee/lower leg	87 (23.7)	54 (16.9)	11 (13.8)
Skin burn	8 (2.2)	18 (5.6)	10 (12.5)
Shoulder/upper arm	22 (6.0)	12 (3.8)	2 (2.5)
Lower back/lumbar spine	11 (3.0)	11 (3.4)	4 (5.0)
Neck/thoracic	13 (3.5)	6 (1.9)	2 (2.5)
Elbow/forearm	10 (2.7)	13 (4.1)	3 (3.8)
other	21 (5.7)	27 (8.4)	15 (18.8)
**Types of injury** (n = 746)			
Open wound	147 (40.0)	115 (36.0)	23 (28.8)
Burn/abrasions	33 (9.0)	47 (14.7)	22 (27.5)
Sprains/strains/dislocations	55 (15.0)	31 (9.7)	9 (11.3)
Fracture	48 (13.0)	33 (10.3)	2 (2.5)
Bruise/contusions	30 (8.2)	24 (7.5)	8 (10.0)
Cut/lacerations	32 (8.7)	43 (13.4)	11 (13.8)
Amputation	11 (3.0)	20 (6.3)	2 (2.5)
**Causes of injury** (n = 724)			
Slips/falls	128 (34.9)	89 (27.8)	14 (17.5)
Burn/explosions	37 (10.0)	55 (17.2)	26 (32.5)
Caught in machinery or equipment	36 (9.8)	60 (18.8)	8 (10.0)
Struck by objects	49 (13.4)	47 (14.7)	3 (3.8)
Overexertion	69 (18.8)	30 (9.4)	9 (11.3)
Foreign bodies	29 (8.0)	25 (7.8)	10 (12.5)

## Data Availability

The original contributions presented in this study are included in the article. Further inquiries can be directed to the corresponding authors.
